# Variability of forced vital capacity in progressive interstitial lung disease: a prospective observational study

**DOI:** 10.1186/s12931-020-01524-8

**Published:** 2020-10-19

**Authors:** Tobias Veit, Michaela Barnikel, Alexander Crispin, Nikolaus Kneidinger, Felix Ceelen, Paola Arnold, Dieter Munker, Magdalena Schmitzer, Jürgen Barton, Sanziana Schiopu, Herbert B. Schiller, Marion Frankenberger, Katrin Milger, Jürgen Behr, Claus Neurohr, Gabriela Leuschner

**Affiliations:** 1 Department of Internal Medicine V, Ludwig-Maximilian University Munich, Marchioninistrasse 15, 81377 Munich, Germany; 2 Comprehensive Pneumology Center (CPC-M), Ludwig-Maximilian University, and Helmholtz Center Munich, Member of the German Center for Lung Research (DZL), Munich, Germany; 3 IBE - Institute for Medical Information Processing, Biometry and Epidemiology, Ludwig-Maximilian University Munich, Munich, Germany; 4 Department of Pneumology, Asklepios Fachkliniken Muenchen-Gauting, Academic Teaching Hospital of the University of Munich, Gauting, Germany; 5 Department of Pneumology and Respiratory Medicine, Hospital Schillerhoehe, Academic Teaching Hospital of the University of Tuebingen, Gerlingen, Germany

**Keywords:** Interstitial lung disease, Idiopathic pulmonary fibrosis, Home spirometry, Forced vital capacity, Variability, Disease progression

## Abstract

**Background:**

Fibrotic interstitial lung disease (ILD) is often associated with poor outcomes, but has few predictors of progression. Daily home spirometry has been proposed to provide important information about the clinical course of idiopathic pulmonary disease (IPF). However, experience is limited, and home spirometry is not a routine component of patient care in ILD. Using home spirometry, we aimed to investigate the predictive potential of daily measurements of forced vital capacity (FVC) in fibrotic ILD.

**Methods:**

In this prospective observational study, patients with fibrotic ILD and clinical progression were provided with home spirometers for daily measurements over 6 months. Hospital based spirometry was performed after three and 6 months. Disease progression, defined as death, lung transplantation, acute exacerbation or FVC decline > 10% relative was assessed in the cohort.

**Results:**

From May 2017 until August 2018, we included 47 patients (IPF *n* = 20; non-IPF *n* = 27). Sufficient daily measurements were performed by 85.1% of the study cohort. Among these 40 patients (IPF *n* = 17; non-IPF *n* = 23), who had a mean ± SD age of 60.7 ± 11.3 years and FVC 64.7 ± 21.7% predicted (2.4 ± 0.8 L), 12 patients experienced disease progression (death: *n =* 2; lung transplantation: *n* = 3; acute exacerbation: *n* = 1; FVC decline > 10%: *n* = 6). Within the first 28 days, a group of patients had high daily variability in FVC, with 60.0% having a variation ≥5%. Patients with disease progression had significantly higher FVC variability than those in the stable group (median variability 8.6% vs. 4.8%; *p* = 0.002). Cox regression identified FVC variability as independently associated with disease progression when controlling for multiple confounding variables (hazard ratio: 1.203; 95% CI:1.050–1.378; *p* = 0.0076).

**Conclusions:**

Daily home spirometry is feasible in IPF and non-IPF ILD and facilitates the identification of FVC variability, which was associated with disease progression.

## Background

Interstitial lung disease (ILD) is a term for a group of conditions including idiopathic pulmonary fibrosis (IPF). Fibrotic ILD such as IPF are all characterized by fibrotic destruction of the lung parenchyma but with great heterogeneity with respect to clinical presentation and prognosis. Furthermore, the individual clinical course of fibrotic ILD can be highly variable, presenting as stable, slowly progressive or rapidly progressive disease [1]. Although therapeutic and diagnostic options for ILD have improved in recent years, the prognosis, especially for IPF, still remains poor [2]. Within a few years of diagnosis, patients can experience dyspnea, hypoxemia and respiratory failure [3]. Patients with progressive, fibrotic ILD suffer from major reductions in quality of life and poor survival, similar to certain malignancies [4]. A reduced forced vital capacity (FVC) has been shown to be the most reliable risk factor for disease progression [5, 6]; indeed, in IPF a FVC decline of 5–10% over 6 months is associated with an increased risk of mortality [6].

In addition to variation in the disease course between patients with fibrotic ILD, disease-related symptoms can be highly variable within a patient. An acute respiratory deterioration is for example seen in the event of an acute exacerbation [7, 8], which has been reported to have a one-year incidence of 3.3–11.5% in non-IPF ILD [9, 10], and 14.2–19.0% in advanced IPF [7, 11]. Importantly, acute exacerbations are associated with an increased mortality risk in IPF and non-IPF ILD [8]. As a consequence of the variable respiratory status, hospital-based pulmonary function tests, which are usually performed every three to six months as part of the routine care of ILD, may not fully capture the extent of the disease. It has been shown that in patients with IPF, daily domiciliary spirometry to measure FVC can be highly clinically informative by potentially helping to detect patients at risk for acute exacerbations, or to monitor the effectiveness of novel therapies [12, 13]. In addition, daily domiciliary spirometry offers the opportunity for a more detailed insight into FVC-changes, and of potential disease progression. For example, in lung transplantation recipients, daily spirometry can identify patients with rapid deterioration, especially those at increased mortality risk [14].

Recently, home spirometry was included in a randomized controlled phase II study with fibrotic unclassifiable ILD [15]. Furthermore, the usefulness of home spirometry was studied in ten patients with sarcoidosis and showed good feasibility [16]. Another very recently published study investigated diurnal variations in FVC using home spirometry in patients with different fibrotic ILDs [17]. Beyond that, home spirometry has not been studied in ILD other than IPF, so far, and literature on home spirometry is still limited. Therefore, the aim of this study was to determine feasibility in different types of fibrotic non-IPF ILD and investigate the clinical impact of daily home spirometry in patients with progressive ILD with respect to disease progression.

## Methods

### Study patients

Individuals with a consensus diagnosis of fibrotic ILD (IPF or non-IPF) [18–20], and subjective clinical progression were recruited from May 2017 until August 2018 in the in- and out-patient unit of the Department of Internal Medicine V of the University of Munich. Subjective clinical progression was defined as an increase of dyspnea and/or physical limitations within the last six months. Patients with pulmonary obstruction (forced expiratory volume in 1 s < 70%) were not included. This study was conducted in accordance with the amended Declaration of Helsinki. Our study was approved by the local ethics committee of the University of Munich (UE No. 812–16) and all participants provided signed, informed consent. Patients were followed for six months, or until death or lung transplantation.

### Study design

Patients were asked to perform daily home spirometry for six months. Hospital-based lung function testing (comprising spirometry, plethysmography and gas transfer), 6-min walking distance, and clinical assessments were performed at baseline, after three and six months. Subjects were also asked to fill out the St. George’s Respiratory Questionnaire (SGRQ), the King’s Brief Interstitial Lung Disease (K-BILD) questionnaire and a 10 cm visual analogue scale (VAS) for cough [21–24].

### Spirometry

Study participants were provided with the hand-held spirometer mySpiroSense® (PARI GmbH, Starnberg, Munich), which conforms with international standards [25]. The spirometer measures FVC by means of a turbine volume transducer and provides a digital read out registered in litres at pressure saturated water vapor (BTPS) and body temperature. Patients received 30 min of instruction on how to perform spirometry, which was repeated at the three month visit. The individual baseline reference values were obtained by using the best FVC from three technically adequate, supervised forced expiratory maneuvers with mySpiroSense® after patients had demonstrated acceptable technique [25]. Patients were asked to perform, if possible, three spirometry maneuvers at approximately the same time of the day every day, with the best value for each day used for further analysis. Patients were unblinded for their own home spirometry results (digital display). Data were recorded on the spirometer and read out and documented electronically at the three and six month hospital visit. Flow-volume curves were also documented by the spirometer and reviewed to ensure validity. The quality of measurements was assessed based on these flow-volume curves and only measurements with good quality flow-volume curves were included in the study. Adherence was calculated as the number of days with home readings divided by the days enrolled in the study, as described before [13]. 

### Statistical analysis

Continuous variables are presented as the mean ± standard deviation (SD), with categorical variables summarized by frequency and percentage. The Wilcoxon rank sum test was used to compare continuous variables, with chi square tests or fisher’s exact tests used to compare categorial variables. Bland-Altman plots were used to compare hospital-based and home spirometry. Pearson’s correlation coefficient was used to correlate hospital spirometry and home spirometry. The hospital-based FVC value was compared to the mean of seven home spirometry FVCs from the respective week at baseline, three months and six months [12, 26]. Individual FVC variability was calculated using the coefficient of variation (CoV) of all home spirometry values within the first 28 days. Additionally, taking potential disease progression into account, individual FVC variability was analysed over three months using the CoV with detrended data points by fitting a linear regression model on individual patients FVC over time and subtracting the residuals, as described before [26]. Pearson correlation was used to compare FVC CoV of the first 28 days, the following 28 days and the 28 days following the three months visit. A linear regression model was applied using only available values without imputation to calculate individual FVC slopes measured by home spirometry or hospital-based spirometry. Slopes of home and hospital-based spirometry were analysed using the pearson’s correlation coefficient. Disease progression was defined as death due to respiratory failure, lung transplantation, acute exacerbation or hospital-based FVC decline > 10% relative at the three or six months visit. Cox proportional hazard regression analysis was applied to identify associations of FVC variability and disease progression including the covariates baseline FVC, age and gender. The R-function “ctree” of the package “party” was used to define a cutoff value of FVC CoV for survival analysis. The Kaplan–Meier method was used to evaluate progression- and transplant-free survival time in the study population, with a log-rank test used to analyse differences between groups. *P* values < 0.05 were considered statistically significant. Data were analysed using SPSS version 25.0 (IBM SPSS, Armonk, NY) or GraphPad Prism version 8.0 for Mac (GraphPad Software, San Diego, California).

## Results

### Study cohort

**Fig. 1:**
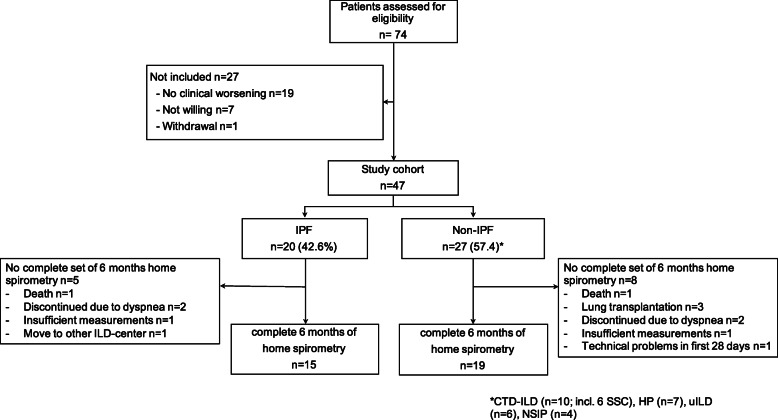
Study cohort. Abbreviations: IPF, idiopathic pulmonary fibrosis; ILD, interstitial lung disease; CTD-ILD, connective tissue disease-related interstitial lung disease; HP, hypersensitivity pneumonitis; uILD, unclassifiable interstitial lung disease; NSIP, non-specific idiopathic pneumonia

Of 74 patients, who were screened for eligibility for the study from May 2017 until August 2018, 47 patients (IPF *n* = 20; non-IPF *n* = 27) were included in the study as depicted in Fig. 1. Non-IPF patients had connective tissue disease-related ILD (*n* = 10), chronic hypersensitivity pneumonitis (*n* = 7), unclassifiable ILD (*n* = 6) or non-specific idiopathic pneumonia (*n* = 4). The IPF cohort was more likely to be male than the non-IPF cohort (80.0% vs. 44.4%; *p* = 0.014), and was older (67.7 ± 7.2 years vs. 59.0 ± 12.7 years; *p* = 0.009) (Table 1). The overall mean ± SD FVC was 63.8 ± 20.9% predicted (2.3 ± 0.8 L), and did not differ between IPF and non-IPF patients. During the study, the majority of patients received medical treatment (IPF 75.0% antifibrotic therapy; non-IPF: 66.7% immunosuppressive therapy).

**Table 1:** Baseline characteristics

	All (***n*** = 47)	IPF (***n*** = 20)	Non-IPF (***n*** = 27)
Age (years)	62.7 ± 11.5	67.7 ± 7.2	59.0 ± 12.7
Gender, male (%)	28 (59.6)	16 (80.0)	12 (44.4)
BMI (kg/m^2^)	27.0 ± 4.5	26.8 ± 4.7	27.2 ± 4.5
Smoking status			
Ex-smoker	24 (51.1)	14 (70.0)	10 (37.0)
Pack years	25.9 ± 20.5	29.6 ± 23.2	20.6 ± 15.6
Lung function			
FVC, % predicted	63.8 ± 20.9	65.6 ± 19.1	62.5 ± 22.4
FVC (L)	2.3 ± 0.8	2.5 ± 0.8	2.2 ± 0.8
TLC, % predicted	69.2 ± 18.8	68.3 ± 16.9	69.9 ± 20.5
TLC (L)	4.2 ± 1.1	4.4 ± 1.2	4.1 ± 1.0
DLCO, % predicted^a^	37.8 ± 15.6	34.5 ± 15.4	40.1 ± 15.7
6MWD (m)^b^	380 ± 121	405 ± 107	364 ± 129
K-BILD	53.2 ± 11.4	50.6 ± 10.6	55.1 ± 11.9
SGRQ^c^	51.5 ± 18.2	57.6 ± 13.0	47.1 ± 20.3
VAS cough (cm)	3.3 ± 2.6	3.8 ± 2.7	2.8 ± 2.4

Data are presented as mean ± standard deviation or number (percentage). Definition of abbreviations: *6MWD* 6-min walk distance, *BMI* body mass index, *DLCO* diffusion capacity for carbon monoxide, *FVC* forced vital capacity, *K-BILD* King’s Brief Interstitial Lung Disease, *SGRQ* St George’s Respiratory Questionnaire, *TLC* total lung capacity, *VAS* visual analogue scale

^a^ *n* = 30 patients (IPF: *n* = 13; non-IPF: *n* = 17). ^b^ *n* = 41 patients (IPF: *n* = 16; non-IPF: *n* = 25). ^c^ *n* = 43 patients (IPF: *n* = 18; non-IPF: *n =* 25)

### Acceptance, adherence and validity of daily home spirometry

Acceptance of home spirometry was high, with only four patients (8.5%; IPF: *n* = 2; non-IPF n = 2) discontinuing within the first week as they were unable to perform daily measurements due to dyspnea. In comparison to the remaining 43 patients, the patients who were not able to perform daily spirometry were significantly older (72.3 ± 7.0 years vs. 61.3 ± 11.4 years; *p* = 0.042). One patient had technical problems with the spirometer within the first 28 days and in two patients more than 50% of the measurements were of poor quality and had to be excluded. Therefore, only 40 patients were included in further analyses (≥50% of measurements in the first 4 weeks). These 40 patients participated for a mean ± SD of 161 ± 38 days (min 28; max 231 days) in the study, performing home spirometry measurements on a mean of 81.8 ± 18.4% days and 98.4 ± 3.5% of these measurements were of good quality. As shown in Figs. 1, 34 patients completed the full set of six months home spirometry. In these patients, adherence was higher within the first three months compared to the second three months (83.5 ± 19.6% vs. 78.4 ± 22.3% of the days; *p* = 0.0086) and did not correlate with patients’ age.

**Fig. 2:**
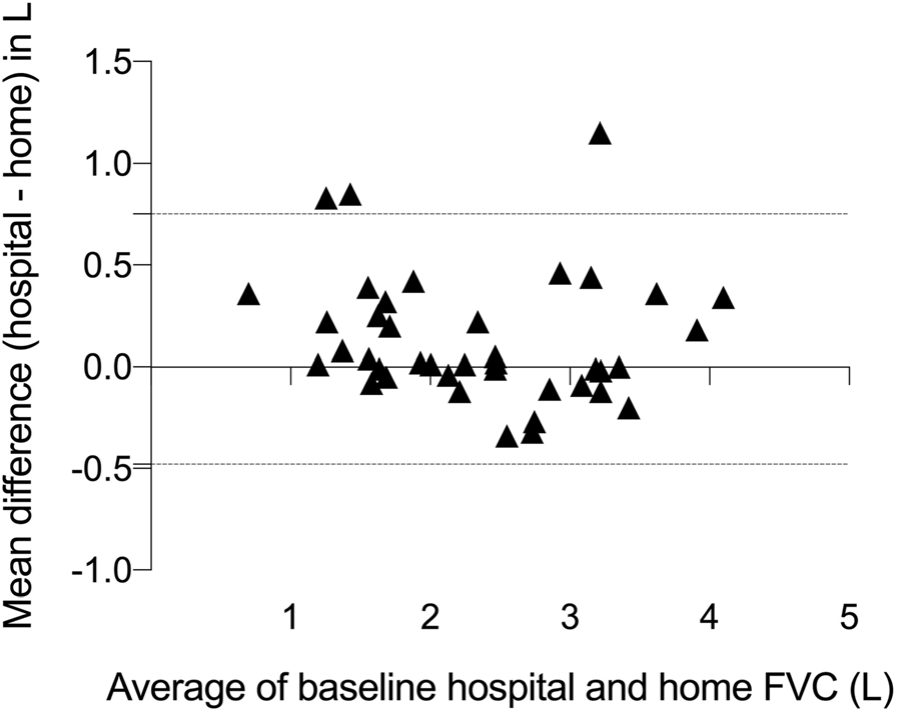
Bland-Altman plot comparing hospital FVC at baseline and home spirometry. Hospital FVC was obtained at the baseline visit; values for home-based FVC are the individual mean of all daily readings within the first seven days of home spirometry. Dashed lines represent the 95% limits of agreement

There was a strong correlation between the baseline hospital FVC value, and the mean of the home FVC measurements over the first seven days (*r =* 0.96; *p* < 0.0001), as well as good overall agreement (bias 0.057 L; 95% limits of agreement − 0.42 to 0.53 L; Fig. 2). The correlation between hospital FVC values and the mean of the home FVC measurements was similarly strong at the three months (*r =* 0.95; *p <* 0.0001) and six months visit (*r =* 0.93; *p <* 0.0001).

A full set of six months home and hospital spirometry was available in 34 patients. Using linear regression analysis, the mean ± SD change in FVC in these patients was − 135.1 ± 303.7 mL (range − 1074.7 to 336.4 mL) measured by home spirometry and − 72.4 ± 227.1 ml (range − 672.1 to 424.0 mL) measured by hospital-based spirometry. The FVC slopes of home and hospital-based spirometry showed a moderate correlation (*r =* 0.441; *p* = 0.009).

### Daily FVC variability

**Fig. 3:**
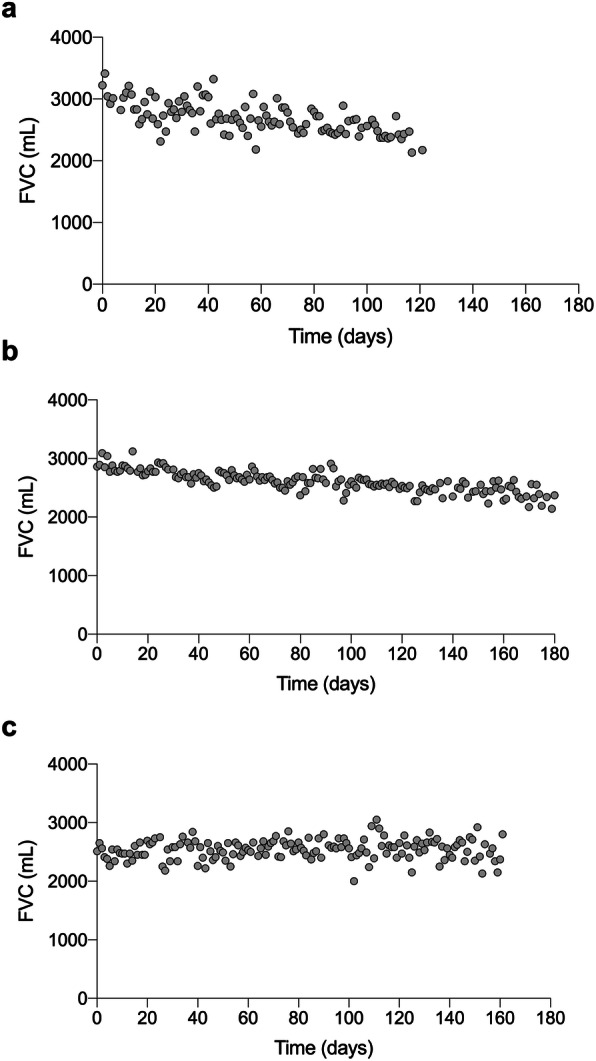
Individual FVC courses in patients with ILD. **a** Patient with non-IPF ILD, who died at day 128. **b** Patient with IPF and FVC decline of 15% relative over six months. **c** Patient with non-IPF ILD and stable lung function

During the study, 12 patients (30.0%; IPF: *n* = 5; non-IPF *n* = 7) had a progression of ILD. Examples of individual FVC courses are shown in Fig. 3. Reasons for disease progression were death (*n* = 2), lung transplantation (*n* = 3 [lung transplantation following acute exacerbation *n =* 2, lung transplantation following FVC decline > 25%: *n* = 1]), acute exacerbation (*n =* 1) and hospital-based FVC decline > 10% (*n* = 6 [FVC decline > 10% at three month visit: *n =* 2; FVC decline > 10% at six month visit *n* = 4]). Of the five IPF patients with disease progression, 80.0% (*n =* 4) were treated with antifibrotic therapy, of the seven patients with non-IPF, 71.4% (*n =* 5) had an immunosuppressive therapy at the time of the study. Although at baseline there were no significant differences in age, FVC % predicted, DLCO, SGRQ or VAS cough between patients with progressive vs. stable disease, the 6MWD (301 ± 140 m vs. 433 ± 89 m; *p* = 0.009) and the K-BILD total score (46.3 ± 8.1 vs. 55.8 ± 12.7; *p* = 0.004) were clearly lower in the progressive group, indicating more physical and subjective wellbeing limitations (Table 2).

**Table 2:** Baseline clinical characteristics of patients with stable or progressive disease

	All (***n*** = 40)	stable (***n*** = 28)	progressive (***n*** = 12)	***p***-value
Age (years)	60.7 ± 11.3	59.6 ± 12.6	63.1 ± 7.6	0.59
FVC, % predicted	64.7 ± 21.7	67.1 ± 21.9	59.2 ± 21.1	0.31
DLCO, % predicted^a^	41.2 ± 19.7	43.1 ± 21.3	36.3 ± 18.4	0.33
6MWD (m)^b^	390 ± 123	433 ± 89	301 ± 140	**0.009**
SGRQ^c^	52.8 ± 18.9	55.2 ± 19.4	48.0 ± 17.7	0.18
K-BILD	52.9 ± 12.2	55.8 ± 12.7	46.3 ± 8.1	**0.004**
VAS cough	3.3 ± 2.6	2.8 ± 2.5	4.4 ± 2.5	0.06

Data are presented as mean ± standard deviation. Definition of abbreviations: *6MWD* 6-min walk distance, *DLCO* diffusion capacity for carbon monoxide, *FVC* forced vital capacity, *K-BILD* King’s Brief Interstitial Lung Disease, *SGRQ* St George’s Respiratory Questionnaire, *VAS* visual analog scale

^a^ *n* = 26 patients (stable: *n* = 19; progressive: *n* = 7); ^b^ *n* = 34 patients (stable: *n* = 23; progressive: *n* = 11); ^c^ *n* = 37 patients (stable: *n =* 25; progressive: *n* = 12);

**Fig. 4:**
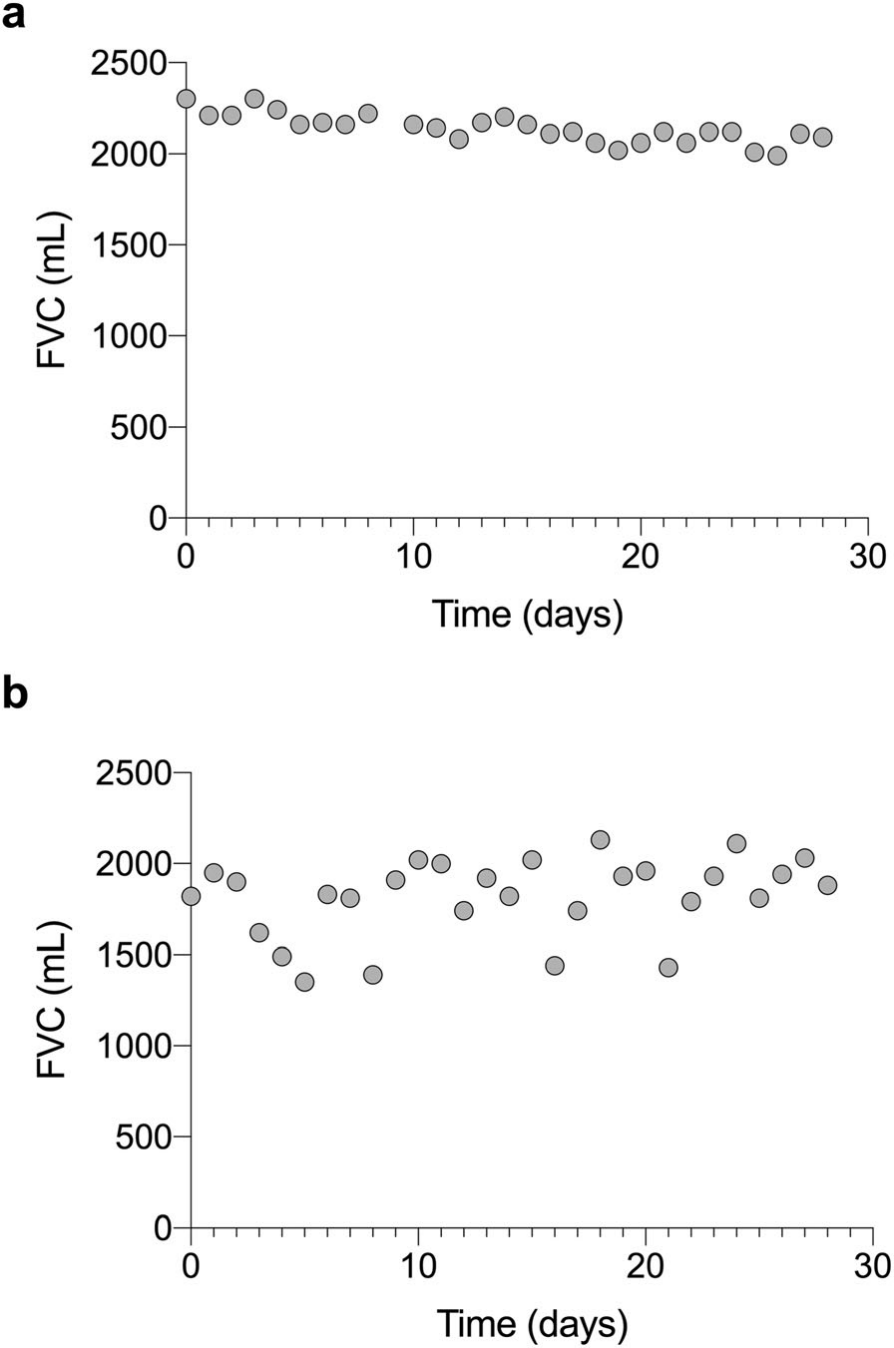
Different variability of daily FVC among representative patients within 28 days. **a** Patient with low variability in FVC (3.7% FVC CoV). **b** Patient with high variability in FVC (11.9% FVC CoV)

**Fig. 5:**
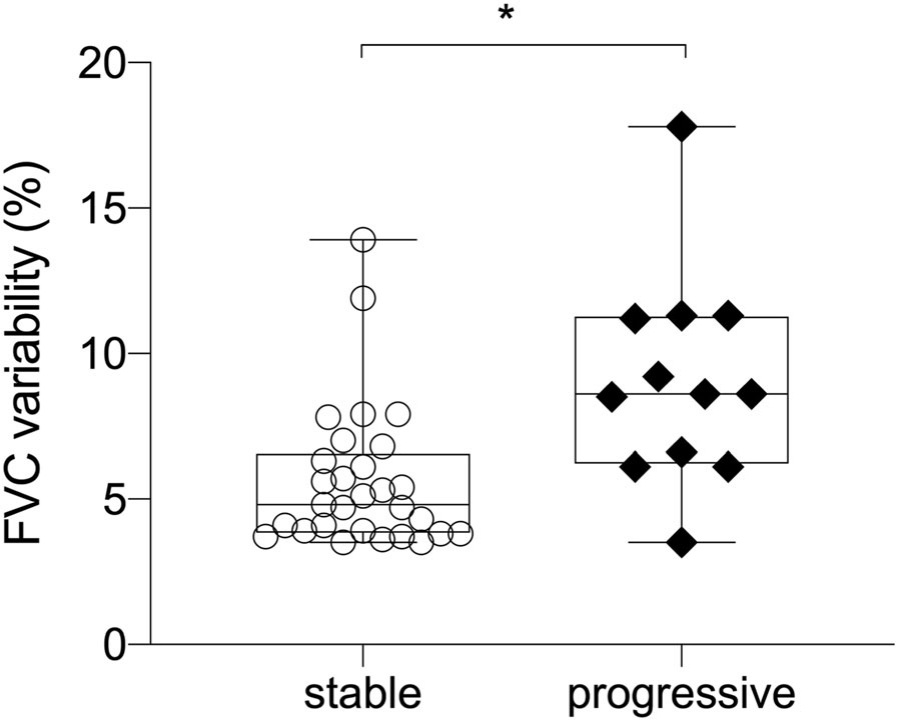
FVC variability in patients with stable and progressive disease. Progressive patients showed a significantly higher FVC variability within the first 28 days of home spirometry in comparison to stable patients (*p* = 0.002)

However, a group of patients had high variability in daily FVC. In the first 28 days (adherence 90.3 ± 12.0%), 60.0% of the patients had FVC CoV ≥5 and 15.0% had FVC CoV ≥10% (Fig. 4). The median of all individual FVC CoVs was 5.9%, ranging from 3.5 to 17.8%. The extent of variation differed clearly between the progressive and stable groups: The median FVC CoV was 8.6% (min 3.5%, max 17.8%) in the progressive group and 4.8% (min 3.5%, max 13.9%) in the stable group (Fig. 5; *p* = 0.002). There was no relationship between CoV and underlying ILD disease entity. Further, the FVC CoV did not differ between patients treated and not treated with antifibrotic therapy (*p* = 0.91).

To evaluate reproducibility and the potential influence of learning effects on FVC variability, a correlation analysis was performed which showed a strong correlation between the FVC CoV of the first 28 days and the following 28 days (*r =* 0.79; *p* < 0.001), as well as a strong correlation between the FVC CoV of the first 28 days and the 28 days following the three months visit (*r =* 0.82; *p <* 0.001). There was no significant difference between the mean ± SD FVC CoV of the first 28 days and the following 28 days (first 28 days: 6.7 ± 3.3; following 28 days: 6.1 ± 3.0; *p* = 0.40). In patients, for whom the FVC CoV following the three months visit was available (*n* = 34), there was also no significant difference between the FVC CoV in the first 28 days and the CoV following the three months visit (first 28 days: 6.5 ± 3.3; 28 days following three months visit: 6.2 ± 2.9; *p* = 0.63). Furthermore, to assure that variability was not driven by short-term lung function deterioration, the FVC CoV and the slope of FVC within the first 28 days were correlated and showed no relationship (*r =* − 0.26; *p* = 0.11).

To assess whether FVC variability can also be observed over a longer observation period, the CoV was further analysed over three months with detrended data points. FVC variability over three months showed a strong correlation with the FVC variability over 28 days (*r =* 0.936; *p* < 0.001). Also, over three months, patients with disease progression had significantly higher FVC variability compared to stable patients (8.4 ± 3.2% vs. 5.5 ± 2.5%; *p* = 0.002).

To see whether patients with disease progression had also higher FVC variability in hospital-based spirometry we further analysed the 34 patients, who had a full set of six months home and hospital spirometry. Of these, seven had a disease progression. The CoV with detrended data points of the three hospital FVCs (baseline, three months and six months) did not significantly differ between the progressive and the stable patients (4.2 ± 3.9% vs. 2.6 ± 2.8%; *p* = 0.391).

### Disease progression

**Fig. 6:**
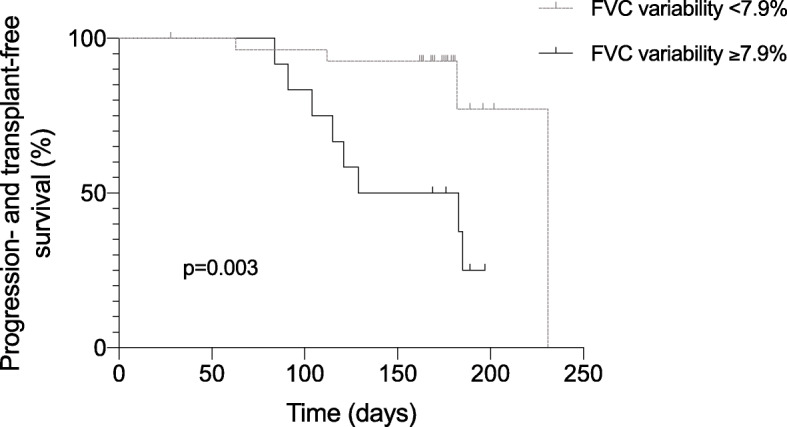
Progression- and transplant-free survival in patients with low and high FVC variability. Based on the optimal cut-off of 7.9%, patients with high FVC variability (≥7.9%) had significantly shorter progression- and transplant-free survival compared to patients with low FVC variability (< 7.9%; *p* = 0.003)

FVC variability over 28 days was independently associated with disease progression (HR 1.203; 95% CI: 1.050–1.378; *p* = 0.0076) while the covariates baseline FVC, age and gender were no significant predictors in the regression analysis in this cohort (Table 3). The optimal cutoff to differentiate between patients with low and high variability was found to be 7.9%. A progress of ILD was found in 14.3% (*n* = 4) of the patients with low variability (< 7.9% rel.) and in 66.7% (*n* = 8) of the patients with high variability (≥7.9% rel.). In the Kaplan-Meier survival analysis, while the six months progression- and transplant-free survival rate was 77.2% in patients with low variability (< 7.9% rel.), it was 37.5% in patients with high variability (≥7.9% rel.; *p* = 0.003; Fig. 6). FVC variability over three months was also a significant predictor for disease progression in the regression analysis with the covariates baseline FVC, age and gender (HR: 1.290; 95%-CI: 1.013–1.643; *p* = 0.039).

**Table 3:** Cox proportional hazard regression analysis assessing the effect on disease progression

	Hazard Ratio	95%-CI	***p***-value
Age	1.036	0.951–1.130	0.416
gender	0.655	0.160–2.682	0.556
Baseline FVC, % predicted	0.997	0.954–1.041	0.876
FVC variability	1.203	1.050–1.378	**0.0076**

FVC variability was assessed over 28 days

Definition of abbreviations: *CI* confidence interval, *FVC* forced vital capacity

## Discussion

The clinical course of ILD can be highly variable, and given the unfavorable prognosis of progressive, fibrotic ILD, it is important to identify predictive factors. Recently, it has been shown that daily home spirometry can provide important information about the clinical course in patients with IPF [12]. However, studies on the usefulness in non-IPF ILD are still limited. Our study focused on home spirometry in patients with IPF and various fibrotic non-IPF ILD, and showed that over one month, FVC can vary greatly in these patients. Furthermore, we demonstrated in the present study that FVC variability was associated with disease progression. Daily home spirometry had a high degree of acceptance, although 8.5% of patients struggled with daily spirometry due to dyspnea and 6.4% had technical problems or poor quality of measurements.

While hospital-based pulmonary function tests cannot be performed on a daily basis, home spirometry allows us to get a more frequent and deeper insight into the respiratory status of our patients, including FVC variability. As shown earlier, the phenomenon of daily spirometric variability seems to occur more often in patients with lung disease than in healthy controls [27]. Recently, daily FVC variability has also been shown in fibrotic ILDs [17]. The authors of this study identified a diurnal variation with higher FVC values in the morning compared to the afternoon. In line with our observations, two other studies described FVC variability of 0.04 to 0.39 L over 28 days of home spirometry in patients with IPF [12, 28]. Indeed, these analyses were only used to evaluate within-subject reproducibility of measurements and were not analysed for their correlation with disease progression [12, 28]. Johannson et al. also observed high variability in FVC in patients with IPF [13], although with home spirometry performed on a weekly basis, and again, variability was not studied in relation to clinical outcome. Interestingly, Russell et al. described a CoV of FVC of 4.96% (range 2.06–20.9%) in IPF patients, which matches our findings [12]. As FVC variability could potentially be caused by factors like spirometry technique (including learning effects), cough, disease progression, mucus production or infection we assessed the relationship between CoV of the first 28 days and the CoV of the second 28 days as well as the CoV of the 28 days following the three months visit and identified a good individual reproducibility. Although the FVC CoV was slightly higher within the first 28 days in comparison to the following 28 days and the 28 days following the three months visit, these differences were not significant. Therefore, in our study, the influence of learning effects (spirometry technique) on the FVC variability appears to be small. Moreover, there was no relationship between CoV and deterioration of lung function within 28 days. Interestingly, our finding that increased variability is associated with disease progression holds true when the variability is assessed over an extended period of time using the CoV over three months with detrended data points [26].

Our results raise the question of whether FVC variability might be a previously unknown predictor for disease progression. In fact, in the past, it has been shown several times that baseline FVC is the most reliable predictor of progression in IPF [5, 6, 29–31]. In our study, however, FVC variability was the only significant predictor for ILD disease progression. It should be noted, that for a major effect of other variables (e.g. baseline FVC) the patient number in our study may have been too small. The observed FVC variability could be interpreted as representation of recurrent subclinical FVC deterioration. We speculate that in a diseased lung, such as ILD, with physiological changes, respiratory capacity can vary on a daily basis as a sign of a vulnerable lung. In chronic diseases with limited mobility, such as advanced ILD, more frequent doctor appointments with pulmonary function tests are often not feasible. Detecting patients at risk for disease progression might allow us to monitor or initiate new medical therapies earlier or evaluate patients for lung transplantation. This further emphasizes the potential utility of domiciliary spirometry in ILD, especially with respect to clinical decision making. In the future, telemedicine will play an important role in patient care. Recently, a pilot study investigated real-time wireless home spirometry in IPF and found it feasible [28].

An important challenge in the future is that adherence to home spirometry among our patients decreased significantly over time. While the median adherence within the first 28 days was 90%, over six months this dropped to 81%. Interestingly, this was not influenced by a small number of patients with very poor adherence, since at the end of the study, only 41% still had an adherence of > 90%. Of note, our spirometer did not include any programmed reminders which might have had a positive influence. Nevertheless, the phenomenon of decreasing adherence occurs even with only weekly measurements [13]. Currently, however, parameters like FVC variability can only be assessed through home spirometry. Therefore, tools have to be developed to minimize barriers and motivate patients to perform FVC measurements on a regular basis, as provided by Moor et al. [28]. Furthermore, as a possible outlook for the future, in clinical practice, it might be sufficient to do daily domiciliary assessments for a limited period of time (for example after diagnosis or one month per year) as part of standard care. Finally, a significant challenge in the future is the interpretation of FVC slopes derived from home spirometry over an extended period of time, for example in clinical trials. In our study, slopes of home and hospital spirometry showed a moderate correlation. Overall, home spirometry detected greater changes in FVC than hospital spirometry. In 2019, the data of a large phase II trial with progressive unclassifiable ILD were published, where the mean change in FVC measured by home spirometry was the primary endpoint [15]. Unfortunately, due to technical problems and physiologically implausible measurements, the data of the primary endpoint could not be sufficiently analysed. Therefore, Maher et al. clearly stated, that further assessments are required, before home spirometry can be used regularly in clinical trials [15]. Very recently data of a randomized controlled trial investigating the benefit of a home monitoring program in terms of improved health related quality of life and medication use for patients with IPF was published [26]. In this study by Moor et al. home spirometry was used, including automated e-mail reminders when spirometry was not performed. Over six months, the authors showed a high adherence rate to home spirometry and comparable slopes of home and hospital spirometry. Given these positive results, programmed reminders and real-time feedback indeed might be a helpful tool in the future to improve quality of home spirometry measurements.

The results of our study should be interpreted in view of the study design and its limitations, which include a single-centre setting and a limited number of patients. The small number of patients included in the study makes it difficult to draw definitive conclusions at this point. Our observations on FVC variability must therefore be interpreted rather as hypothesis-generating and still require validation in separate (multi-centric) studies in the future. Nevertheless, the results of our study should be considered relevant and important as this is the first study to identify daily FVC variability as a potential prognostic factor in ILD. Furthermore, not all patients were able to perform daily spirometry due to dyspnea. Still, this was a real-life experience and our study was designed to include patients with progressive ILD, who presumably would have a high dyspnea burden – and despite this, there was a high level of acceptability of daily spirometry. In line with this finding, in another IPF cohort 13% of patients were unable to perform home spirometry [13]. However, our study does suggest that home spirometry may not be feasible in all patients with ILD.

## Conclusion

In conclusion, we demonstrated that in ILD, FVC can vary greatly on a daily basis. Moreover, higher FVC variability was associated with disease progression in ILD. Although home spirometry was feasible and accepted in the majority of patients, daily use can be challenging and might not be feasible for all patients in the long term. Nonetheless, home spirometry (even when performed over a limited period) could help to detect patients at risk of disease progression, and so might be beneficial for clinical decision making and in ILD research in the future. Future studies are needed to further determine the role of FVC variability and to evaluate whether it can be used as a biomarker of disease progression in patients with fibrotic ILD.

## Data Availability

The datasets generated and analysed during the current study are not publicly available due privacy of our patients but are available from the corresponding author on reasonable request.

## Abbreviations

CoV: Coefficient of variation
FVC: Forced vital capacity
ILD: Interstitial lung disease
IPF: Idiopathic pulmonary fibrosis
K-BILD: King’s Brief Interstitial Lung Disease Questionnaire
SD: Standard deviation
SGRQ: St. George’s Respiratory Questionnaire
VAS: Visual analogue scale
